# The accuracy of MRI in the detection of Lumbar Disc Containment

**DOI:** 10.1186/1749-799X-3-46

**Published:** 2008-10-02

**Authors:** Bradley K Weiner, Rikin Patel

**Affiliations:** 1 Weill Cornell Medical College, USA; 2 Department of Orthopaedic Surgery, The Methodist Hospital, Houston, Texas, USA; 3 Institute for Orthopaedic Research and Education, The Methodist Hospital Houston, Texas, USA

## Abstract

**Background:**

MRI has proven to be an extremely valuable tool in the assessment of normal and pathological spinal anatomy. Accordingly, it is commonly used to assess containment of discal material by the outer fibers of the anulus fibrosus and posterior longitudinal ligaments. Determination of such containment is important to determine candidacy for intradiscal techniques and has prognostic significance. The accuracy of MRI in detecting containment has been insufficiently documented.

**Methods:**

The MRI's of fifty consecutive patients undergoing open lumbar microdiscectomy were prospectively evaluated for disc containment by a neuroradiologist and senior spinal surgeon using criteria available in the literature and the classification of Macnab/McCulloch. An independent surgeon then performed the surgery and documented the actual containment status using the same methods. Statistical evaluation of accuracy was undertaken.

**Results:**

MRI was found to be 72% sensitive, 68% specific, and 70% accurate in detecting containment status of lumbar herniated discs.

**Conclusion:**

MRI may be inaccurate in assessing containment status of lumbar disc herniations in 30% of cases. Given the importance of containment for patient selection for indirect discectomy techniques and intradiscal therapies, coupled with prognostic significance; other methods to assess containment should be employed to assess containment when such alternative interventions are being considered.

## Introduction

Magnetic resonance imaging has proven to be an indispensable tool for the orthopaedic spine surgeon. Its value in assessing normal lumbar anatomy, internal disc chemistry and architecture, features of lumbar spine degeneration, and in diagnosing herniated lumbar discs has been well documented [1]. Accordingly, MRI is often used for assessing containment of herniated lumbar discs by the fibers of the outer annulus fibrosis or posterior longitudinal ligament. The determination of such containment has become rather important given (a) the continued surge in the use of indirect techniques of lumbar discectomy and newer intradiscal therapies and (b) recent evidence that containment status may have a direct impact on prognosis with or without surgical treatment for sciatica.

Techniques such as percutaneous suction discectomy, laser discectomy, chemonucleolysis, and newer intradiscal techniques [2-5] rely upon a single unifying theory; that by decreasing the pressure centrally within the disc, a central flow of more peripheral nuclear material will follow, thus allowing an indirect nerve root decompression. This theory relies upon disc containment to allow central nuclear flow and clinical studies using indirect discectomy techniques have demonstrated inferior results in the treatment of extruded discs [6-8].

Additionally, Carragee[9] and others [10-15] have demonstrated that outcomes of both operative and non-operative treatments for sciatica secondary to lumbar disc herniations are associated with containment (or lack thereof) and qualitative characteristics of outer annular/posterior longitudinal ligament fibers. Hence, containment status appears prognostically important and has a place in the provision of informed consent.

That said, studies evaluating the accuracy of MRI in the detection of lumbar disc containment have been insufficient. While MRI has been shown sensitive and specific for detecting lumbar disc sequestration[16,17], to our knowledge only three previous articles pertaining to containment have been published, all of which are contained in the older literature. Grenier[18] evaluated the MRI findings commensurate with posterior longitudinal ligament disruption in cadaveric specimens. But found, when applying such findings in the prospective part of his study, MRI able to detect containment in only seven of eleven surgically documented cases. Silverman[19] (via retrospective chart review) evaluated MRI criteria for containment and found an intact low signal intensity line representing the posterior longitudinal ligament, small disc herniation size and absence of free disc fragments to be rather poor predictors of containment. Kim [20] reported reasonable sensitivity/specificity in the detection of containment; but the surgeon was not blinded to the pre-operative reports, was not independent (was a reader), and Gadolinium enhancement was added retrospectively in cases which were disputed.

The objective of the current paper was to address the accuracy of MRI in detecting containment using *more solid methodological controls*. A neuroradiologist and a senior spine surgeon with extensive experience with indirect techniques prospectively evaluated 50 consecutive MR images of herniated lumbar nucleus pulposes to predict disc containment and, subsequently, these findings were independently compared with the intra-operative findings during open microdiscectomy allowing statistical evaluation of MRI regarding its ability to detect disc containment.

## Methods

The MR images of fifty consecutive patients meeting the following criteria were included in this study: (1) The patient presented with a unilateral radicular syndrome involving a single lumbar nerve root. (2) The MRI demonstrated a single level lumbar disc herniation commensurate with the patient's history and physical exam. (3) The MRI was performed at our center using a 1.5 -T superconductive unit (Siemens Magnetom). (4) Both T1 and T2 sagittal and axial images were available and were of high quality. (5) The patient was refractory to conservative care and underwent open discectomy with subsequent resolution of symptoms. And (6) all surgeries were performed by an independent surgeon using the operative microscope taking great care to assess containment.

The age of the patients ranged from 28 to 68 years and averaged 43 years. There were 35 males and 15 females. Three herniations occurred at the L3-L4 disc space, 28 at L4-L5, and 19 at L5-S1. All surgeries were performed within a one year time interval.

**Figure 1:**
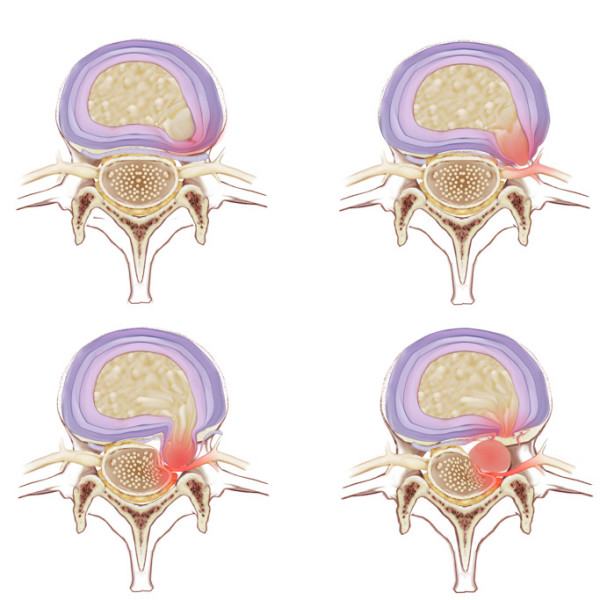
Classification of discal pathology.

The MR images of the included fifty patients were read independently and blinded to all clinical and surgical information by two readers; a neuroradiologist specializing in MRI and a senior spine surgeon with extensive experience using indirect techniques of lumbar discectomy. The readers independently classified the herniations using the system of Macnab and McCulloch[21] as follows (Figure 1):

(1) Disc Protrusion: A bulging disc with intact annular and posterior longitudinal ligamentous fibers.

(2) Subannular Extrusion: Disrupted inner annular fibers with intact outer annular fibers and intact posterior longitudinal ligament.

(3) Transannular Extrusion: Disrupted annulus fibrosis and posterior longitudinal ligament with intact tail of disc material extending into disc space.

(4) Sequestration: Free fragment without tail extending into disc space. The first two categories are considered "contained" and the last two "non-contained".

These readings were then compared with the intra-operative findings. All surgeries were performed by one surgeon (a leader in spinal microsurgery who was not one of the pre-op readers in the study) using an open technique of discectomy with lighting and visualization enhanced by the operative microscope. The disc herniations were classified intra-operatively using the same classification scheme noted above and the surgeon was blinded to all of the MRI readings.

Statistical breakdown of the MRI readings into four groups (true and false positives and true and false negatives) allowed assessment of sensitivity, specificity, and accuracy using standard calculations[22] as well as ROC (Receiver Operating Characteristic) graphic evaluation. Correlation between the two readers of MRI's was assessed using the Kappa coefficient/statistics.

## Results

**Figure 2:**
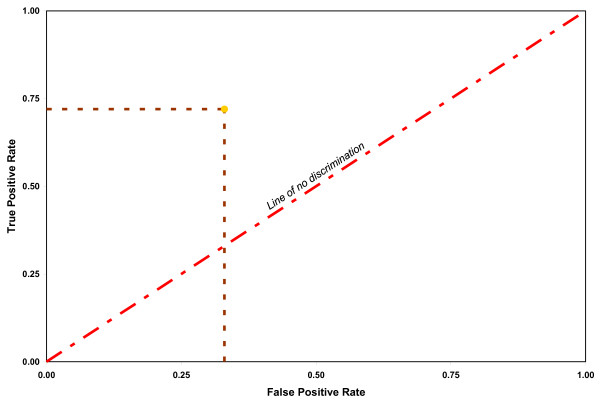
The ROC graph for MRI detection of discal containment.

Intra-operative pathology of disc protrusion was documented in 13 cases, subannular extrusions in 16 cases, transannular extrusions in 17 cases, and sequestrations in four cases. These were then grouped into contained (protrusion, subannular) and non-contained (transannular, sequestration) since surgical decisions regarding indirect techniques of discectomy would be based upon these two broad categories. MR imaging in these 50 patients produced eight false negative and 21 true positive diagnoses of containment. There were seven false positive and 14 true negative diagnoses (Table 1). Overall, sensitivity was 72%, specificity 68%, and accuracy 70% for MR imaging in detecting containment of herniated lumbar discs. The true positive rate was .72 and the false positive rate was .33. The ROC graph is depicted in Figure 2.

**Table 1:** A 2 × 2 contingency table for MRI Disc Containment

	Contained Herniation at Operation	Non-Contained Herniation at Operation
Contained Herniation on MRI	**21 ***(TP*)	**7 ***(FP)*
Non-Contained Herniation on MRI	**8 ***(FN)*	**14 ***(TN)*

The interobserver agreement was .90 with a Kappa of .796 at 95% confidence. Outlying cases were resolved by consensus between the two readers.

## Discussion

Patient selection for the less invasive, indirect techniques of lumbar discectomy and intradiscal therapies remains problematic. Both theoretically and clinically, inferior results with these techniques are noted when disc extrusion has occurred beyond the boundaries of the outer annulus and posterior longitudinal ligament[6-8]. Clinical symptoms and signs cited as suggestive of disc containment include increased pain with sitting but relief with recumbency and lack of pain during straight-leg raising of the uninvolved side (i.e., no crossover). While some surgeons use such clinical findings as indications for indirect discectomy techniques, most feel that MRI evidence of containment is crucial. Higher intensity of sequestered disc fragments has been noted on T2 images[16,17]. Fries[23] and Postacchini[24] have demonstrated that herniations greater than 50% of the AP thecal sac diameter are likely to be non-contained. Grenier[18] in an anatomic study, found MRI to be rather accurate in detecting outer annular and posterior longitudinal ligament fibers as low signal intensity lines and this finding has become accepted as indicating containment when the line is continuous.

These findings would suggest, then, that an MRI image noting similar signals between herniated disc material and that material remaining within the disc space, small to moderate herniation size, and intact low intensity signal representing outer annular or posterior longitudinal ligament fibers indicates disc containment. Hence, patients with such an MRI are often considered candidates for an indirect technique of discectomy.

The current study, however, has demonstrated that such MRI findings are not consistently predictive of containment. Of 29 surgically documented contained discs, MRI images suggested eight of these to be non-contained; and of 21 surgically documented non-contained discs, MR images suggested containment of seven discs using the above criteria as guidelines.

The source of the problem appears to lie in the evaluation of the outer annular and posterior longitudinal ligament fibers. Grenier[18] demonstrated a low signal intensity line on MRI which correlated with outer annular and posterior longitudinal ligament fibers in anatomic/cadaveric specimens. He was able to visualize these structures in all specimens and to differentiate them from inner annular fibers and the dura. Interestingly, however, in the prospective portion of his study he was able to detect surgically verified containment of discal material in only seven of eleven cases using MRI and Silverman[19], in a separate (and, again, older) retrospective study noted above, found similar findings.

These findings, coupled with those in our current study, raise several points: First, the anatomic portion of the above noted study was performed on three specimens, each of which was taken from young, fresh cadavers. Conclusions drawn from images of young, healthy ligamentous structures may not correlate with those in degenerated/herniated discs. Pathophysiological changes in the nucleus, inner annulus, outer annulus and posterior longitudinal ligaments may cloud the differentiation of these structures on MR imaging. Second, cadaveric tissue (especially in the anterior spinal canal) may differ from the tissues in vivo given the lack of nutritional supply. Third, an inflammatory process may accompany the disc herniations, further confounding the images. Fourth, particular MRI cuts/sections may miss annular fissures or tears. Fifth, a change in status in containment may occur between the time of MRI and the time of treatment. And sixth, poor spatial resolution of MRI resulting from volume averaging may lead to inaccurate depiction of ligamentous integrity. The sum of these factors (coupled with extrinsic factors including those involved in the actual readings of the MRI) results in a less than satisfactory delineation between containment and non-containment of herniated lumbar discs.

In summary then, we have found MRI to be 72% sensitive, 68% specific, and 70% accurate in detecting containment of herniated lumbar discs. Given that the success of indirect techniques of lumbar discectomy, other intradiscal therapies, and prognosis following herniation all rely upon accurate assessment of disc containment; MRI alone may provide insufficient or inaccurate information upon which to base surgical/technical decisions in about of 30% of cases. Other methods to determine containment (when considering indirect techniques) should be employed/considered [25].

## Competing interests

The authors declare that they have no competing interests.

## Authors' contributions

BW conceived and performed the study. RP assisted with Tables/Figure and statistical analysis.
